# Moral courage efficacy among medical students: associations with environmental professionalism, empathy attitudes, and communication self-efficacy

**DOI:** 10.3389/fmed.2026.1812249

**Published:** 2026-06-26

**Authors:** Keren Michael, Nick Farajev, Orit Karnieli-Miller

**Affiliations:** 1 Department of Human Services, Department of Social Work, Community Health Promotion Research Center, The Max Stern Yezreel Valley College, Yezreel Valley, Israel; 2 Department of Medical Education, Gray Faculty of Medical and Health Sciences, Tel Aviv University, Dr. Sol Amsterdam Dr. David P. Schumann Chair in Medical Education, Tel Aviv, Israel

**Keywords:** communication, empathy, moral courage, professionalism, self-efficacy

## Abstract

**Background:**

Moral courage efficacy is the belief in one’s perceived ability to take an active stand or action in the face of others- and self-wrongdoing or moral injustice, despite a risk of negative consequences. Although moral courage is important for enhancing patient safety and quality of care, there is limited research on moral courage efficacy and its environmental and personal contributing factors to guide educational interventions. The current study examined the complex relationships between environmental professionalism, empathy attitudes, communication self-efficacy, and moral courage efficacy.

**Methods:**

The sample included 178 clinical medical students who had completed online self-reported questionnaires. Data were analyzed via Pearson’s correlation tests and Hayes’s regression-based PROCESS macro.

**Results:**

The study findings showed that environmental professionalism was directly associated with moral courage efficacy toward others (*β* = 0.40) and toward the self (*β* = 0.44). Empathy attitudes was indirectly associated with both moral courage efficacy dimensions through communication self-efficacy (*β* = 0.08 and *β* = 0.09, respectively).

**Conclusion:**

The findings highlight the need for educational interventions to focus on organizational (environmental) and personal (enhancing empathy and communication self-efficacy) levels to enhance moral courage efficacy. Intervening on both levels may help create a safer space, increased motivation, and perceived ability for students to disclose others’ and their own wrongdoing, thus potentially promoting a safer and more moral medical environment.

## 1 Introduction

Moral courage is the willingness to take an active stand or action when facing wrongdoing or moral injustice, despite adversity and risk of adverse consequences (1–5). Moral courage relates both to addressing others’ wrongdoing (e.g., another physician’s medical error) as well as admitting one’s own wrongdoing (6). Articulation of these faults can result in negative consequences from the environment, e.g., being disgraced, excluded, attacked, punished, or poorly evaluated, which can become barriers to morally courageous behavior (3). Although these barriers are challenging and difficult to overcome, enhancing the understanding and development of moral courage is essential. Moral courage is critical for improving patients’ safety by promoting a “speaking up” culture, ensuring quality of care, and providing treatment according to professional values (7, 8).

Furthermore, moral courage is important to individuals’ well-being (9), allowing them to deal with ethical challenges in the workplace (6, 10), and act according to their beliefs. Either failing to act in a morally courageous way or feeling incapable of acting according to one’s morals may lead to an experience of moral distress (5), i.e., *“*…*a negative emotional state that results when a person feels inhibited from addressing a situation felt to be ethically problematic due to external constraints…”* (11).

Studies exploring moral courage (in healthcare, focused mainly on nurses) (12, 13) identified inhibiting factors, including fear of repercussions (2, 3, 7), bystander effect of sharing responsibility, and hoping that others would act (2, 14). Other studies identified promotors of moral courage, including role models who accept morally courageous actions (7, 15).

A factor that warrants exploration is one’s feelings of being capable of acting morally courageously, i.e., moral courage efficacy (16). This refers to Bandura’s concept of self-efficacy, i.e., individuals’ belief regarding how well they can execute the actions required to deal with prospective situations and accomplish desired goals (17, 18). Self-efficacy is important as people are most likely to engage in activities they believe they can handle. In contrast, they will feel deterred from performing actions they lack the confidence to face (17). Self-efficacy regarding a particular skill is an important motivational factor in applying such capabilities (19). Thus, students with high moral courage efficacy may be more prone to morally courageous behavior than those with low moral courage efficacy.

As self-efficacy is important, further research is needed to identify extrinsic and intrinsic factors contributing to medical students’ (MS) moral courage efficacy. Our study examined how students’ perceptions of the attitudes and behavioral norms of professionalism in their medical environment, their personal empathy attitudes, and communication self-efficacy are associated with moral courage efficacy.

### 1.1 Environmental professionalism

As moral courage is a social act with possible social consequences, it may be influenced by the norms of the environment (20). Throughout their career, MS undergo a socialization process in which they acquire the set of values, attitudes, and behaviors of the professional culture (21). They observe role models’ behavior in the medical environment and learn from their explicit (“formal curriculum”) and implicit (“hidden curriculum”) teachings (21–23). These teaching settings set the rules for professionalism in an environment, i.e., defining physicians’ expected and unexpected behavior.

These teachings can encourage professional behavior, i.e., placing patients’ interests above their own and aspiring to altruism, accountability, excellence, service, integrity, and respect for others (24). In contrast, they can reinforce problematic, unprofessional behaviors, e.g., dishonesty and lack of self-awareness (22, 25, 26). Earlier studies indicated that MS are sometimes exposed to unprofessional behavior in the clinical environment (22, 27). Exposure to such an environment has powerful impacts on learners (28), including their present and future ability to act according to their moral values when faced with unethical or unprofessional situations (11, 29, 30).

Within clinical training, the relevance of environmental professionalism to moral courage efficacy may also be understood in relation to medical students’ and trainees’ relatively low-power positions within hierarchical healthcare organizations. This hierarchy is perceived as a barrier to speaking up about unprofessional or ethically problematic behavior (7). Speaking up about concerns may depend on the presence of a psychologically safe environment, i.e., a shared belief that the team is safe for interpersonal risk taking, including asking questions, admitting mistakes, and raising concerns (31, 32).

Thus, the professional environment may contribute to moral courage efficacy in at least two ways: first, by modeling desirable conduct through the formal and hidden curricula (21, 23, 33, 34), and second, by conveying whether raising concerns, admitting uncertainty, or disclosing errors is legitimate, expected, and safe within the clinical learning environment (7, 31, 32). Although the professional environment may play an important role in learners’ perceived capability to act morally courageously, this association has received limited empirical attention.

### 1.2 Empathy

Individual and personal factors, such as empathy attitudes, may also be relevant to moral courage efficacy. Empathy is described as the ability to take the perspective of others, understand their feelings or experiences from their point of view, and therapeutically communicate this understanding (35–37). Empathy is an important skill for healthcare professionals as it is related to altruism and altruistic behavior (38). Empathy may increase students’ sensitivity to patient vulnerability and morally troubling situations, thereby strengthening the motivation to respond on behalf of others (38). In that way, empathy may promote and drive the motivation to act courageously on others’ behalf (e.g., to help a mistreated patient) (1).

However, research distinguishes other-oriented empathic concern from self-oriented personal distress, suggesting that whereas empathic concern may promote prosocial and altruistic behavior, excessive personal distress or overidentification with patients may lead to emotional burden, withdrawal, or avoidance (38, 39). Furthermore, theoretical and empirical literature suggests that empathy is multidimensional and may not uniformly translate into effective moral action.

Empathy is commonly conceptualized as including cognitive, affective, and behavioral components, such as perspective-taking, emotional attunement, and the communication of this understanding (39). Thus, empathy attitudes may be insufficient for moral courage efficacy unless students also perceive themselves as capable of communicating concerns clearly, respectfully, and effectively in emotionally demanding situations. Accordingly, while empathy attitudes may be relevant to moral courage efficacy, their role among medical students requires further empirical investigation, particularly in relation to students’ perceived ability to communicate concerns in challenging clinical situations.

### 1.3 Communication self-efficacy

the belief in one’s capability to apply high-quality interpersonal and communication skills (40), may also contribute to moral courage efficacy, as high-quality communication has been shown to improve physicians’ and students’ social skills (41), interpersonal relationships, and exchange of information (42). Communication skills may be important to moral courage, as the morally courageous act itself is often presented as a communication intervention, usually by “speaking up.” (4, 7) Thus, communication competence, or belief about one’s ability (i.e., self-efficacy), has the potential to be associated with and lead to higher levels of willingness and belief in one’s ability to act morally courageously.

Due to the role of communication in moral courage (4, 7), communication self-efficacy may serve as a theory-based mediator in the association between environmental professionalism or empathy and moral courage efficacy. Without communication skills, even in a highly professional learning environment, people may feel incompetent to speak up. Furthermore, as elaborated above, although empathy is critical to identifying a breach of professionalism or moral wrongdoing and to feeling the urge to do something about it, the ability to act on this feeling may require a sense of competence in one’s communication skills (35). Though communication self-efficacy was associated with empathy (40), earlier studies did not directly explore these associations with moral courage efficacy.

### 1.4 The current study

Thus, our study elaborates on the existing research regarding the complex relationships between environmental professionalism, empathy attitudes, communication self-efficacy, and moral courage efficacy. The novelty of the current study lies in examining moral courage efficacy, rather than moral courage behavior or general moral courage, as a distinct self-efficacy construct among clinical medical students. Moreover, the study integrates environmental and personal variables within a single model and examines communication self-efficacy, which may be developed in training, as a potentially derived mechanism linking these variables to moral courage efficacy. Understanding these associations has an educational benefit in guiding interventions to enhance moral courage efficacy during medical school training.

### 1.5 Hypotheses

*H*_1_: Environmental professionalism, empathy attitudes, communication self-efficacy, and moral courage efficacy will be positively intercorrelated.

*H*_2_: Communication self-efficacy will mediate the associations between environmental professionalism or empathy attitudes and moral courage efficacy.

## 2 Methods

### 2.1 Participants and procedure

This quantitative cross-sectional study was conducted among 178 MS aged 24–37 (*M* age = 29.79, *SD* = 2.66). Approximately 57% were female. All were MS from Israeli universities during their clinical clerkships, distributed across various wards and hospitals. Thus, they were potentially exposed to different environmental professionalism norms, attitudes, and behaviors. The inclusion criteria were: being enrolled as a medical student in an Israeli university, being in the clinical stage of medical training, including clinical clerkships or internship, and providing informed consent. No additional exclusion criteria were applied. Data were collected between March and August 2022. Participants were recruited via convenience sampling. The questionnaires were distributed electronically via students’ personal email accounts and in closed groups on social media. The participants completed the forms using the Qualtrics platform. The university IRB approved this study. The participants’ detailed demographic characteristics are presented in Table 1.

**Table 1:** Demographic characteristics of the study participants (*N* = 178).

Variable	Category	Frequency	Percent
Gender	Male	76	43.2
	Female	100	56.8
Birth country	Israel	165	93.8
	Other	11	6.3
Religion	Jewish	165	94.3
	Christian	2	1.1
	Muslim	6	3.4
	Other	2	1.1
Family status	Single	45	25.7
	In a relationship	57	32.6
	Married	73	41.7
Number of children	0	128	71.1
	1	28	16.5
	2 or more	14	12.4
Economic status	Below average	24	13.6
	Average	59	33.5
	Above average	93	52.9
Educational institute	Tel Aviv University	146	82
	Technion (Haifa)	3	1.7
	The Hebrew University (Jerusalem)	13	7.3
	Ben-Gurion University (Beer Sheba)	10	5.6
	Bar-Ilan university (Safed)	6	3.4
Program and stage	Four-year (3rd year)	4	2.3
	Four-year (4th year)	13	7.4
	Four-year (internship)	6	3.4
	Six-year (4^th^ year)	20	11.4
	Six-year (5th year)	41	23.4
	Six-year (6th year)	71	40.6
	Six-year (internship)	20	11.4

### 2.2 Measurements

All questionnaires used in the current study were based on previously published instruments. For each instrument, the original source and available psychometric information are provided below. The questionnaires were administered to participants in Hebrew. When a validated Hebrew version was not available, the items underwent a translation–back-translation procedure and were reviewed by the research team to ensure conceptual clarity and relevance to the Israeli medical education context. The two scales developed by the first and last authors (16, 40) are provided in the Supplementary materials in both English and Hebrew.

Moral courage efficacy was measured using an 8-item questionnaire (16) consisting of two dimensions: The first focused on moral courage related to others’ wrongdoing (items 1–4; e.g., “Capable of intervening when a physician behaves immorally toward a patient”) and the second focused on moral courage related to the self, i.e., enables the individual to admit some kind of wrongdoing or need for feedback (items 5–8; e.g., “Capable of revealing my lack of knowledge and of asking when I am in doubt”). All items were rated on a 5-point Likert scale (1 = to a very small extent, 5 = to a very great extent) and calculated by averaging the answers on each dimension, with higher scores representing higher moral courage efficacy. Internal reliability on the original scale (16) was *α* = 0.80 for the “others” dimension and *α* = 0.84 for the “self” dimension. In the current study, the Cronbach’s alpha coefficients were 0.82 and 0.84, respectively.

Environmental professionalism was measured using a 12-item scale (43). This questionnaire can be evaluated as a total score or as consisting of three dimensions: Excellence (items 1–5; e.g., “During this rotation I have individuals whom I consider role models”); Honor/Integrity (items 6–9; e.g., “I have observed my residents lie to a patient”), and Altruism/Respect (items 10–12; e.g., “I have observed residents making derogatory statements about other medical specialty groups or other health care workers”). All items were rated on a 10-point Likert scale (1 = strongly disagree; 10 = strongly agree) and calculated by summing up the answers (after recoding items 6–12), with a higher total score representing higher levels of professionalism. Internal reliability on the original scale (43) was *α* = 0.71. In the current study, the Cronbach’s alpha was 0.91.

Empathy attitudes were measured using the 20-item S-JSPE questionnaire (44) examining students’ orientation toward empathy in a patient–physician relationship (e.g., “Physicians’ understanding of their patients’ feelings and the feelings of their patients’ families is a positive treatment factor”). All items were rated on a 7-point Likert scale (1 = strongly disagree; 7 = strongly agree) and calculated by averaging the answers (after recoding items 8, 10, 12), with higher scores representing more positive attitudes toward empathy. Internal reliability on the original scale (44) was *α* = 0.80. In the current study, the Cronbach’s alpha was 0.88.

Communication self-efficacy was measured using a 15-item questionnaire (40), examining perceived self-confidence to apply good communication in the medical encounter (e.g., “Capable of controlling/restraining myself in situations that are emotionally challenging for me”). All items were rated on a 5-point Likert scale (1 = to a very small extent; 5 = to a very large extent) and calculated by averaging the answers, with higher scores representing higher communication self-efficacy. Internal reliability on the original scale (40) was *α* = 0.84. In the current study, the Cronbach’s alpha was 0.86 (For psychometric characteristics of all measurements, see Table 2).

**Table 2:** Psychometric characteristics and correlations among the study variables.

Variable	1	2	3	4	5
1. Moral courage efficacy (others)	--	0.57***	0.43***	0.11	0.26**
2. Moral courage efficacy (self)		--	0.47***	0.10	0.28***
3. Environmental professionalism			--	0.11	0.16*
4. Empathy attitudes				--	0.42***
5. Communication self-efficacy					--
*M* (*SD*)	2.63 (0.91)	3.95 (0.82)	84.05 (22.95)	4.14 (0.48)	4.02 (0.46)
*Range*	1.00–5.00	1.00–5.00	25.00–118.00	1.85–4.95	1.85–4.95
*α*	0.82	0.84	0.91	0.88	0.86

*p < 0.05; **p < 0.01; ***p < 0.001. M, mean; SD, standard deviation; Range-minimum–maximum; α, Cronbach’s alpha.

### 2.3 Data analysis

We used IBM-SPSS (version 25) to analyze the data. First, we used descriptive statistics to calculate frequencies, central tendencies, and the dispersion of the study variables. Then, we calculated Cronbach’s alphas for the research tools’ structure and inter-reliability estimates (after all the required transformations). Next, we used Pearson’s correlation tests to examine the first hypothesis regarding bivariate associations among the study variables. To examine the second hypothesis regarding theory-based indirect associations through communication self-efficacy, we used Hayes’s regression-based PROCESS macro (version 3.3). We examined the significance of the indirect associations by calculating 5,000 bootstrapped samples to estimate the 95% bias-corrected confidence intervals (CIs) of indirect effects of the predictor on the outcome through the mediator (45).

Model diagnostics were examined for the regression models underlying the PROCESS analyses. These included assessment of multicollinearity (VIF), residual independence (Durbin-Watson), homoscedasticity (Breusch-Pagan), residual normality (Shapiro–Wilk), and influential observations (Cook’s distance). Additionally, effect sizes for each regression equation were estimated using R^2^ and Cohen’s f^2^, interpreted according to Cohen’s conventions (46). The defined significance level was generally set to 5% (*p* < 0.05).

## 3 Results

Our study examined the complex relationships between environmental professionalism, empathy attitudes, communication self-efficacy, and moral courage efficacy among MS. H1 examined bivariate correlations among the study variables. Table 2 demonstrates that, as expected, environmental professionalism had moderate-strong positive associations with moral courage efficacy toward others (*r* = 0.43, *p* < 0.001) and the self (*r* = 0.47, *p* < 0.001). Similarly, communication self-efficacy had a moderate positive association with both dimensions of moral courage efficacy (*r* = 0.26, *p* < 0.01 and *r* = 0.28, *p* < 0.001, respectively). However, empathy was not associated with moral courage efficacy dimensions (*r* = 0.11, *p* > 0.05 and *r* = 0.10, *p* > 0.05, respectively). Thus, H1 was partially supported.

Before interpreting the regression-based PROCESS analyses, model diagnostics were examined across the regression equations. All VIF values were below 1.25, Durbin-Watson statistics ranged from 1.92 to 2.02, and no influential observations were identified based on Cook’s distance. Breusch-Pagan tests indicated heteroscedasticity across the regression equations, and a minor deviation from residual normality was observed in one moral courage efficacy outcome model (W = 0.983, *p* = 0.027). Because the indirect effects were evaluated using bootstrap confidence intervals based on 5,000 samples, these deviations are unlikely to meaningfully affect the interpretation of the reported indirect associations; nevertheless, the regression-based findings should be interpreted with appropriate caution.

**Figure 1:**
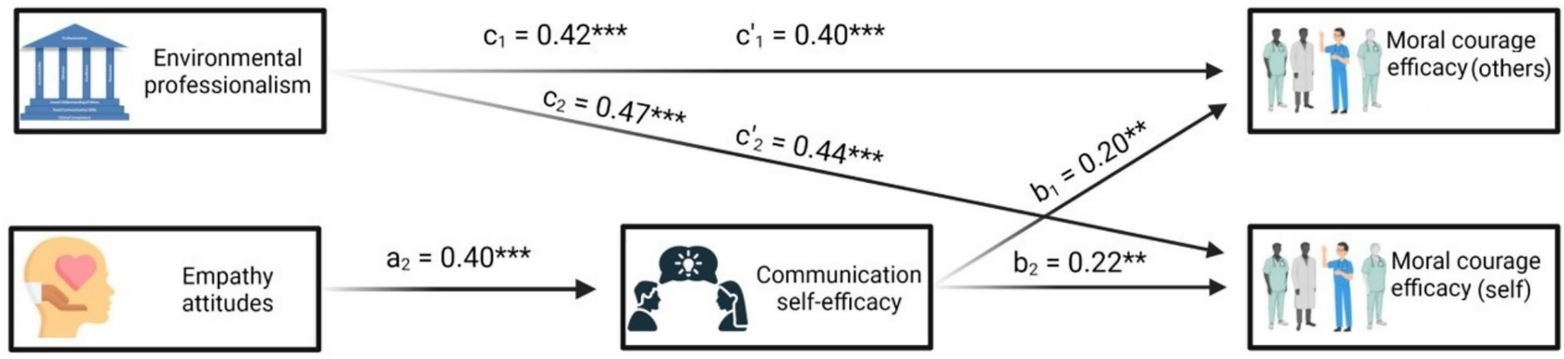
The study model: the association between environmental professionalism, empathy attitudes, communication self-efficacy, and moral courage efficacy. *p < 0.05; **p < 0.01; ***p < 0.001. Only paths with significant *β* were depicted in this flowchart.

H2 examined indirect associations among the study variables. As shown in Table 3 and Figure 1, no significant indirect associations were found between environmental professionalism and either dimension of moral courage efficacy through communication self-efficacy: moral courage efficacy toward others (*β* = 0.02; LLCI, ULCI = −0.00, 0.00) or moral courage efficacy toward the self (*β* = 0.02; LLCI, ULCI = −0.00, 0.00). Instead, we found significant total and direct effects of environmental professionalism. In this way, environmental professionalism was associated with moral courage efficacy toward others (*β* = 0.42; LLCI, ULCI = 0.01, 0.02) and self (*β* = 0.47; LLCI, ULCI = 0.01, 0.02; paths c_1_ and c_2_, respectively), even after controlling for these associations (*β* = 0.40; LLCI, ULCI = 0.01, 0.02 and *β* = 0.44; LLCI, ULCI = 0.01, 0.02; paths c’_1_ and c’_2_, respectively).

**Table 3:** Total, direct, and indirect effects of environmental professionalism and empathy attitudes on moral courage efficacy through communication self-efficacy.

Predictor	B	*SE*	β	*T*	*LLCI, ULCI*
Dependent variable: moral courage efficacy (other)					
Environmental professionalism (path c_1_ = total effect)	0.02	0.00	0.42	6.19	0.01, 0.02
Empathy attitudes (path c_3_ = total effect)	0.15	0.13	0.08	1.13	−0.11, 0.40
Environmental professionalism (path c_’1_ = direct effect)	0.02	0.00	0.40	5.92	0.01, 0.02
Empathy attitudes (path c_’3_ = direct effect)	−0.00	0.14	−0.00	−0.02	−0.28, 0.27
Communication self-efficacy (path b_1_)	0.38	0.14	0.20	2.64	0.1, 0.67
Environmental professionalism > Communication self-efficacy (path a_1_b_1_ = indirect effect)	0.00	0.00	0.02	--	−0.00, 0.00
Empathy attitudes > Communication self-efficacy (path a_2_b_1_ indirect effect)	0.15	0.06	0.08	--	0.04, 0.29
Dependent variable: moral courage efficacy (self)					
Environmental professionalism (path c_2_ = total effect)	0.02	0.00	0.47	6.89	0.01, 0.02
Empathy attitudes (path c_4_ = total effect)	0.10	0.12	0.06	0.83	−0.13, 0.33
Environmental professionalism (path c_’2_ = direct effect)	0.02	0.00	0.44	6.62	0.01, 0.02
Empathy attitudes (path c_’4_ = direct effect)	−0.05	0.12	−0.03	−0.43	−0.30, 0.19
Communication self-efficacy (path b_2_)	0.39	0.13	0.22	2.97	0.13, 0.64
Environmental professionalism > Communication self-efficacy (path a_1_b_2_ = indirect effect)	0.00	0.00	0.02	--	−0.00, 0.00
Empathy attitudes > Communication self-efficacy (path a_2_b_2_ indirect effect)	0.15	0.07	0.09	--	0.03, 0.29
Mediator: communication self-efficacy					
Environmental professionalism (path a_1_)	0.00	0.00	0.11	1.65	−0.00, 0.01
Empathy attitudes (path a_2_)	0.39	0.07	0.40	5.84	0.26, 0.52

B, unstandardized beta; SE, standard error for the unstandardized beta; β, standardized beta; T, t-test statistic; LLCI–ULCI, lower limit of the confidence interval–upper limit of the confidence interval.

The results supported the hypothesized indirect association between empathy attitudes and moral courage efficacy through communication self-efficacy. In path a (independent variable→ mediator), we found that empathy was positively associated with communication self-efficacy (*β* = 0.40; LLCI, ULCI = 0.26, 0.52; path a_2_). Additionally, in path b (mediator→ dependent variable), we found that communication self-efficacy was positively associated with both dimensions of moral courage efficacy (*β* = 0.20; LLCI, ULCI = 0.1, 0.67 and *β* = 0.22; LLCI, ULCI = 0.13, 0.64; paths b_1_ and b_2_, respectively). When examining path ab (mediator’s indirect effect), the CIs did not contain zero. Thus, significant indirect associations were found between empathy attitudes and both dimensions of moral courage efficacy through communication self-efficacy: moral courage efficacy toward others (*β* = 0.08; LLCI, ULCI = 0.04, 0.29) and moral courage efficacy toward the self (*β* = 0.09; LLCI, ULCI = 0.03, 0.29; paths a2b1 and a2b2, respectively).

To further contextualize the magnitude of these findings, effect sizes were examined for each regression equation underlying the PROCESS analyses. The predictors accounted for 18.6% of the variance in communication self-efficacy (R^2^ = 0.186, f^2^ = 0.229), 22.6% of the variance in moral courage efficacy toward others (R^2^ = 0.226, f^2^ = 0.293), and 26.3% of the variance in moral courage efficacy toward the self (R^2^ = 0.263, f^2^ = 0.357). According to Cohen’s conventions (46), the mediator and moral courage efficacy toward others models showed medium effect sizes, whereas the moral courage efficacy toward the self-model showed a large effect size.

## 4 Discussion

In view of the importance of moral courage for enhancing patient safety and quality of care, its relevance to individuals’ well-being, and the lack of understanding of contributing factors (47), our study examined the associations between environmental professionalism, empathy attitudes, communication self-efficacy, and moral courage efficacy among MS. The findings demonstrated that environmental professionalism was associated with moral courage efficacy toward others and the self, but was not mediated by communication self-efficacy. The association between empathy attitudes and moral courage efficacy was mediated by communication self-efficacy. This pattern suggests that environmental professionalism may be a particularly relevant contextual correlate of moral courage efficacy, whereas the role of empathy attitudes appears to be more indirect and modest in magnitude, operating through communication self-efficacy.

The findings indicating a direct association between environmental professionalism and moral courage efficacy emphasized the environment’s impact on professional identity formation, where MS learn by observation which norms and behaviors are acceptable (34, 48–50). Being a part of a highly professional environment, where professionalism is not only discussed but also acted upon (46, 51), where the norms encourage medical staff’s professional behavior of placing the patients’ interests above their own and aspiring to integrity and respect for others (24) may convey a message to students that immoral actions are unacceptable and can be addressed. Such environments may support the belief that addressing these issues through morally courageous actions is legitimate and even encouraged.

An organization’s ethical climate, i.e., the set of formal and informal shared perceptions that shape expectations for ethical behavior in the workplace (52) can affect the ethical courage of its employees (47). Our study suggests that learners who perceived their environment as practicing professionalism also reported greater perceived capability (and perhaps more responsibility) to behave with moral courage. Another possible explanation is that students in highly professional environments may be exposed to role models who demonstrate exemplary morally courageous behavior. These professionals, for example, may disclose their errors and challenges, or act assertively by speaking up when they encounter others’ mistakes. These professionals may even promote moral courage by inviting students to ask questions or express concerns (53). Thus, students in these environments may learn through role modeling and imitation (49), potentially strengthening their perceived own capability to act in the same manner.

Understanding this mechanism is important, as it implies a possible interrelated reciprocal effect of environmental professionalism, enhancing moral courage efficacy and, hopefully, morally courageous behavior. A highly professional environment may provide conditions that are associated with greater moral courage that, in turn, will help improve the professional environment. Obviously, this relationship in an unprofessional environment can have the opposite effect; those who remain silent become part of the unprofessional culture (54).

The fact that communication self-efficacy did not mediate this association is interesting. Even when one possesses communication self-efficacy, sharing criticism and being morally courageous still involves taking a risk. Studies reported that individuals were willing to expose their vulnerability and take interpersonal risks only in psychologically safe environments (32, 55, 56) and in a climate in which they felt encouraged to do so (7). Our findings imply that learners might infer that speaking up is not welcome in an unprofessional environment toward patients. They may perceive this environment as a less psychologically safe space where transparent communication of a sensitive issue is far more challenging, even for individuals with high communication self-efficacy.

As expected, empathy attitudes were indirectly associated with moral courage efficacy through communication self-efficacy. The role of empathy is related to awareness and the ability to identify a problem, evoking the potential motivation and the urge to do something about it. However, empathy alone or attitudes toward empathy are insufficient. This interpretation may also explain why empathy attitudes were not directly associated with moral courage efficacy in the current study: empathy may heighten moral sensitivity and concern, but its translation into perceived capability to act may depend on students’ communication self-efficacy and on their ability to regulate emotional distress in demanding clinical contexts. However, potentially, in some situations, emotionally overwhelming empathic responses or overidentification with patients may even hinder action by increasing avoidance or withdrawal.

To translate empathic attitudes into morally courageous action, individuals need to take an active stand (1), applying their communication skills (7). The ability to communicate sensitive information calmly and assertively is essential (57). This understanding supports earlier studies showing that possessing moral sensitivity and identifying ethical problems are not enough to facilitate morally courageous acts (58). If the perceived ability to share one’s concern is lacking, the perceived ability to act to bring about change is limited. The gap between knowing what is right and being unable to act on it may lead to frustration and even moral distress (29). Hence, there is an implied need to strengthen students’ motivation and practical capability to exercise moral courage, as well as to impart the relevant practical communication skills to do it.

### 4.1 Limitations and future research

Our study sheds light on the concept of moral courage efficacy and its associated environmental and personal variables. However, some limitations deserve attention. First, participants were recruited via convenience sampling of MS in one country, undermining the external validity and generalizability of the findings. This sampling approach may also have introduced selection bias, as students who chose to participate may differ from those who did not in their interest in professionalism, empathy, communication, or moral courage.

Second, the study used a cross-sectional design and, therefore, cannot determine the temporal sequence of the variables or establish causal relationships among them. This limitation is particularly important in relation to the mediation analyses, which should be interpreted as theory-based indirect associations rather than evidence of causal mediation. Alternative directions of association are also plausible.

Third, all variables were measured using self-report questionnaires administered at a single time point. This may increase the risk of common method bias and social desirability bias, particularly because the study addressed morally and professionally valued attitudes and perceived capabilities. Although internal consistency was assessed for each scale, additional data quality indicators, such as response time, attention checks, or response-pattern analyses, were not available.

Fourth, the study did not include several potentially relevant control variables, such as burnout, stress (16), personality traits, or prior exposure to ethical dilemmas, which may be related to the observed associations among the study variables. Future studies may benefit from assessing them and their relationship to empathy, as well as using Davis’s empathy measure, which distinguishes between empathic concern and personal distress, where excessive personal distress or overidentification with patients may lead to emotional burden, withdrawal, or avoidance, which may influence whether individuals engage with, withdraw from, or avoid morally challenging situations (38, 59).

Finally, the questionnaire used to assess communication self-efficacy (40) focused on a student–patient relationship. The communication skills required for this relationship may differ from those that are applicable to moral courage in communications with colleagues or supervisors. A more general questionnaire examining either communication competence (60), communication skills related to teamwork, or assertiveness (57) may be further examined.

## 5 Conclusion and practical implications

Our study depicts a direct, positive association between environmental professionalism and moral courage efficacy, suggesting that organizational culture may be an important contextual factor related to students’ perceived capability to act morally courageously in the complex, high-stakes medical environment. This relationship may have worrying implications in unprofessional environments where the perceived ability to act morally courageously is lower and thus less likely to prevent additional breaches of patient safety and quality care. Furthermore, the findings also suggest that empathy attitudes and communication self-efficacy may jointly be relevant to MS’ moral courage efficacy. Thus, these findings may inform interventions that address both environmental and personal factors.

Medical education interventions would benefit from focusing on faculty development by encouraging professionalism in patient care (61) and facilitating the conditions for employees (and, in our case, learners) to act morally courageously (47). At the same time, environmental interventions could focus on the students, helping them develop an awareness of how unprofessional behavior affects them and how to reduce its negative impact (61). Parallel, interventions should focus on enhancing empathy, identifying moral dilemmas, and providing communication training to impart the tools/manners to share concerns, be assertive, and speak up. In summary, intervening on both environmental and personal levels may help create a safer space and increase motivation and perceived ability for students to disclose others’ and own wrongdoing, thus potentially promoting better patient care.

## Data Availability

The raw data supporting the conclusions of this article will be made available by the authors, without undue reservation.

## Author’s note

Study findings were presented at the 15th Israeli National Institute for Health Policy (NIHP) conference, May, 2023; and the 9th International Conference on Public Health (ICOPH), August, 2023, virtual.
