# Supplementary material for: Moral courage efficacy among medical students: associations with environmental professionalism, empathy attitudes, and communication self-efficacy
Source: Front Med (Lausanne). 2026 Jun 26;13:1812249. doi: 10.3389/fmed.2026.1812249 (PMC13349757; doi:10.3389/fmed.2026.1812249)
Supplement: Supplementary file 1 [file Data_Sheet_1.PDF]

Moral courage efficacy scale

Please indicate the extent to which you currently believe you are capable of performing what is described in each sentence.

"I believe that I am…:"	**To a very small extent**				**To a very Large extent**
1. Capable of intervening when a physician behaves immorally toward a patient	1	2	3	4	5
2. Capable of expressing my feelings of discomfort to a senior physician following his/her problematic behavior toward patients, or following problematic behavior toward patients by another physician in the department	1	2	3	4	5
3. Capable of telling a senior physician if I have identified a concern about an error s/he might have made	1	2	3	4	5
4. Capable of telling a senior physician that I have been asked to perform a task that is against my moral principles	1	2	3	4	5
5. Capable of disclosing my mistakes to a senior physician	1	2	3	4	5
6. Capable of disclosing my lack of knowledge and of asking when in doubt	1	2	3	4	5
7. Capable of saying that I lack the competence expected of me to perform a medical procedure on a patient	1	2	3	4	5
8. Capable of inviting criticism and feedback in various situations	1	2	3	4	5

Communication self-efficacy scale

Please indicate the extent to which you currently believe you are capable of performing what is described in each sentence.

"I believe that in a physician–patient encounter, I am…:"	**To a very small extent**				**To a very Large extent**
1. Capable of letting the patient to tell his/her story	1	2	3	4	5
2. Capable of building a trustful relationship with the patient	1	2	3	4	5
3. Capable of identifying the patient's emotional state	1	2	3	4	5
4. Capable of addressing the patient's emotional state and not merely his/her physical state	1	2	3	4	5
5. Capable of noticing the patient's nonverbal cues/behavior	1	2	3	4	5
6. Capable of acting in a caring manner toward the patient's emotional state	1	2	3	4	5
7. Capable of making the patient feel I am not judging him/her	1	2	3	4	5
8. Capable of addressing a situation with understanding when the patient prefers a different treatment than the one I recommend	1	2	3	4	5
9. Capable of controlling/restraining myself in situations that are emotionally challenging for me	1	2	3	4	5
10. Capable of being friendly, and never being either crabby or rude to the patient	1	2	3	4	5
11. Capable of explaining any technical medical terms in plain language	1	2	3	4	5
12. Capable of dealing with the patient's emotions	1	2	3	4	5
13. Capable of asking open-ended and closed-ended questions at a pace that is suited to the patient	1	2	3	4	5
14. Capable of asking the patient sensitive and personal questions	1	2	3	4	5
15. Capable of leading the encounter at a pace that is appropriate to the patient's needs, while keeping within the time constraints of a medical encounter	1	2	3	4	5
