# Supplementary material for: Moral courage efficacy among medical students: associations with environmental professionalism, empathy attitudes, and communication self-efficacy
Source: Front Med (Lausanne). 2026 Jun 26;13:1812249. doi: 10.3389/fmed.2026.1812249 (PMC13349757; doi:10.3389/fmed.2026.1812249)
Supplement: Supplementary file 2 [file Data_Sheet_2.PDF]

שאלון מסוגלות לאומץ מוסרי

אנא סמן/י עד כמה את/ה מאמין/ה שאת/ה מסוגל/ת לבצע היום את מה שמתואר בכל משפט.

"אני מאמין/ה שאני..."	**במידה מועטה מאוד**				**במידה רבה מאוד**
1. מסוגל/ת להתערב כשרופא מתנהג באופן לא מוסרי למטופל	1	2	3	4	5
2. מסוגל/ת לשתף רופא בכיר בתחושה של אי נוחות עקב התנהגות בעייתית שלו או של רופא אחר במחלקה כלפי מטופלים	1	2	3	4	5
3. מסוגל/ת לומר לרופא בכיר אם זיהיתי חשש לטעות שלו	1	2	3	4	5
4. מסוגל/ת לשתף רופא בכיר אם התבקשתי לבצע משימה הנוגדת את עקרונות המוסר שלי	1	2	3	4	5
5. מסוגל/ת לחשוף את טעויותיי בפני רופא בכיר	1	2	3	4	5
6. מסוגל/ת לחשוף את אי ידיעותיי ולשאול כשאני בספק	1	2	3	4	5
7. מסוגל/ת לומר שחסרה לי המיומנות המצופה ממני לביצוע פרוצדורה רפואית במטופל	1	2	3	4	5
8. מסוגל/ת להזמין ביקורת ומשוב במצבים שונים	1	2	3	4	5

מסוגלות תקשורת

אנא סמן/י עד כמה את/ה מאמין/ה שאת/ה מסוגל/ת לבצע היום את מה שמתואר בכל משפט.

"אני מאמין/ה שבמפגש רופא-מטופל אני..."	**במידה מועטה מאוד**				**במידה רבה מאוד**
1. מסוגל/ת לתת למטופל אפשרות לספר את סיפורו	1	2	3	4	5
2. מסוגל/ת לפתח יחסי אמון עם המטופל	1	2	3	4	5
3. מסוגל/ת לזהות את המצב הרגשי של המטופל	1	2	3	4	5
4. מסוגל/ת להתייחס למצבו הרגשי של המטופל ולא רק למצבו הפיסי	1	2	3	4	5
5. מסוגל/ת לשים לב להתנהגות הבלתי מילולית של המטופל	1	2	3	4	5
6. מסוגל/ת להתנהג באכפתיות בנוגע למצבו הרגשי של המטופל	1	2	3	4	5
7. מסוגל/ת לתת למטופל תחושה שאיני שופט/ת אותו	1	2	3	4	5
8. מסוגל/ת להתייחס בהבנה למצב בו המטופל מעדיף טיפול אחר מזה שהמלצתי לו	1	2	3	4	5
9. מסוגל/ת לשמור על איפוק במצבים המאתגרים אותי מבחינה רגשית	1	2	3	4	5
10. מסוגל/ת להיות ידידותי/ת ולעולם לא עצבני/ת או גס/ת-רוח כלפי המטופל	1	2	3	4	5
11. מסוגל/ת להסביר את כל המונחים הרפואיים-טכניים בשפה פשוטה	1	2	3	4	5
12. מסוגל/ת להתמודד עם הרגשות של המטופל	1	2	3	4	5
13. מסוגל/ת לשאול שאלות פתוחות וסגורות בקצב המותאם למטופל	1	2	3	4	5
14. מסוגל/ת לשאול את המטופל שאלות רגישות ואישיות	1	2	3	4	5
15. מסוגל/ת להוביל את המפגש בקצב המותאם לצורכי המטופל, תוך שמירה על מגבלות הזמן של מפגש רפואי	1	2	3	4	5
